# Long non-coding RNA-polycomb intimate rendezvous

**DOI:** 10.1098/rsob.200126

**Published:** 2020-09-09

**Authors:** Andrea Cerase, Gian Gaetano Tartaglia

**Affiliations:** 1 Blizard Institute, Barts and The London School of Medicine and Dentistry, Queen Mary University of London, London E1 2AT, UK; 2 Centre for Genomic Regulation (CRG), The Barcelona Institute for Science and Technology, Dr. Aiguader 88, 08003 Barcelona, Spain; 3 Universitat Pompeu Fabra (UPF), 08003 Barcelona, Spain; 4 Institucio Catalana de Recerca i Estudis Avançats (ICREA), 23 Passeig Lluis Companys, 08010 Barcelona, Spain; 5 Department of Biology ‘Charles Darwin’, Sapienza University of Rome, P.le A. Moro 5, Rome 00185, Italy; 6 Department of Neuroscience and Brain Technologies, Istituto Italiano di Tecnologia (IIT), Via Morego 30, 16163, Genoa, Italy

**Keywords:** long non-coding RNAs (lncRNA), Xist RNA, HOTAIR RNA, polycomb-repressive complexes 1/2 (PRC1/2), RNA–protein interaction, RNA secondary structure, phase separation

## Abstract

The interaction between polycomb-repressive complexes 1/2 (PRC1/2) and long non-coding RNA (lncRNA), such as the X inactive specific transcript *Xist* and the HOX transcript antisense RNA (HOTAIR), has been the subject of intense debate. While cross-linking, immuno-precipitation and super-resolution microscopy argue against direct interaction of Polycomb with some lncRNAs, there is increasing evidence supporting the ability of both PRC1 and PRC2 to functionally associate with RNA. Recent data indicate that these interactions are in most cases spurious, but nonetheless crucial for a number of cellular activities. In this review, we suggest that while PRC1/2 recruitment by *HOTAIR* might be direct, in the case of *Xist*, it might occur indirectly and, at least in part, through the process of liquid–liquid phase separation. We present recent models of lncRNA-mediated PRC1/2 recruitment to their targets and describe potential RNA-mediated roles in the three-dimensional organization of the nucleus.

## 1. Polycomb-group repressive complexes

Polycomb-repressive complexes 1/2 (PCR 1/2) are repressive proteins, firstly described in *Drosophila melanogaster*, responsible for the Hox-genes silencing [1], and competing with activating factors such as these from the Thritorax-group proteins [2]. In *Drosophila*, Polycomb complexes are recruited to target genes by recognition of polycomb response elements, also called PREs [3]. While mammals largely lack canonical PREs, CpG islands seem to pay an equivalent role [4]. Polycomb-mediated silencing is essential for several cellular functions, from pluripotency [5] to lineage specification [6] senescence and cancer [7,8]. In mammals, Polycomb complexes come in two main flavours, polycomb-repressive complex 1 (PRC1) and Polycomb-repressive complex 2 (PRC2). Each of these complexes can be further divided into three [9] or six [10] subcomplexes, respectively, depending on the complex composition and cellular function [11,12]. Polycomb complexes are responsible for placing repressive chemical marks on histone tails, regulating chromatin functions. In particular, PRC2 places methyl groups at the lysine 27 of the histone H3 [13], via Ezh2, its catalytic subunit, while PRC1 places mono-ubiquitin moieties at lysine 119/120 of histone H2A [14], via the Ring1A/B catalytic subunits. Polycomb marks on the chromatin, are, in turn, read by Polycomb complexes subunits [15,16] (positive reinforcing loops) and other complexes (readers) to stabilize gene silencing [17]. Both Polycomb complexes are capable of binding RNA, and this function of Polycomb complexes is crucial to ensure correct gene expression [18–20]. While PRC1/2 complexes are thought to have non-catalytic roles in genome architecture (e.g. by organizing the genome in three-dimensional) [21], the catalytic activity of these complexes is critical for polycomb-mediated silencing [22–24]. As the role of these marks has been discussed elsewhere, we refer the reader to other excellent reviews [25,26]. In our review, we focus on the role of RNA and in particular of long-coding RNAs, in the recruitment of these complexes to the chromatin, using the two most studies lncRNAs, Xist and HOTAIR, as models.

## 2. Direct versus indirect binding of polycomb-repressive complexes 1/2 components to Xist and HOTAIR

Long non-coding RNAs (lncRNAs) are RNA molecules longer than 200 bases that lack protein-coding potential [27,28]. They represent a significant portion of the cell transcriptome [29] and work as activators or repressors of gene transcription acting on different regulatory mechanisms [30–32]. lncRNAs can act as scaffolds for protein recruitment [33–40] and behave as guides and/or sponges for titrating RNAs and proteins, influencing transcription at regulatory regions or triggering transcriptional interference [41–43]. In the large *spectrum* of activities, the RNA structure plays a central role and dictates precise functionalities by creating spatial patterns and alternative conformations and binding sites for proteins [44,45]. In this review, we will focus on the two best-studied lncRNAs, *Xist* and *HOTAIR*, to critically discuss what we know about the interaction of PRC1/2 complexes with RNA.

**Figure 1.**
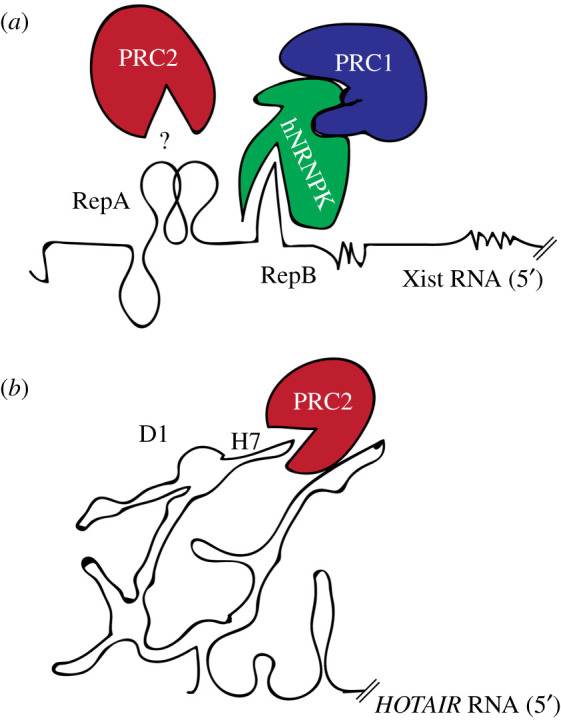
*Xist and HOTAIR interactions with Polycomb proteins*. (*a*) Schematic representation of possible PRC1/2 interactions via the Xist A- [51] and B- [52] repeats (black lines). The question marks indicates the debated interaction of PRC2 with the A-repeat (*b*) Schematic representation of *HOTAIR* interaction at domain 1 (D1), helix 7 (H7) [53] with PRC2 (black lines).

*Xist* is a long non-coding RNA and the master-regulator of X chromosome inactivation (XCI) [46–49]. *Xist* works as a scaffold for the recruitment of repressive complexes on the inactive X chromosome (Xi) [46,50]. As for its structure, six conserved repetitive regions (Rep), named A to F, have been reported to be essential for its function [30,44]. The interaction between *Xist* and PRC1/2 has been studied in detail. In particular, PRC1 has been reported to interact with *Xist* B-repeats and PRC2 with *Xist* A-repeats (see below) (figure 1*a*). In the case of PRC1-*Xist* B repeats, a study from the Heard laboratory showed that a region encompassing the *Xist* B/C-repeat is necessary for PRC1 recruitment [52]. The Brockdorff laboratory mapped this interaction to the B repeat mostly, and proved that HNRNPK, which physically interacts with PRC1, is directly involved in RNA binding (figure 1*a*) [54]. For the PRC2-*Xist* interaction with the A-repeats, there is not agreement in literature. A seminal study from the Lee laboratory has shown that *Xist* A-repeats directly recruits EZH2 via direct interaction with its stem and loops [51]. However, different lines of evidence stemming from developmental studies suggest that *Xist* expression and PRC2 recruitment can be decoupled. In particular, in developing female embryos, *Xist* RNA clouds seems to precede H3K37me3 domains, making a direct interaction unlikely [55,56]. In agreement with these observations, super-resolution microscopy [57] and genetics analysis [58] point towards a non-direct interaction. In particular, Almeida *et al.* suggest that *Xist* attracts PRC2 to the chromatin via the recognition of the chromatin mark placed by PRC1 (i.e. H2AK119ub), in agreement with other models of PRC1/2 recruitment [59,60] (discussed in more details below).

*HOTAIR* [61] is another well-known lncRNA regulating the expression of the HOX genes during development [61]. *HOTAIR* works as a scaffold for the recruitment of the PRC2 members EZH2, SUZ12, and it is also able to act *in trans* to allow the establishment of a repressed chromatin state at the HOX clusters [62,63]. How *HOTAIR* interacts with PRC2 *in vivo* is still debated, an *in vitro* study indicates a direct interaction between HOTAIR and EZH2 at its 5′ [63,64]. In particular, *HOTAIR* interaction with PRC2*,* mapped at the HOTAIR repeat D1 helix 7 (H7) [53], appears to be direct (in the range of 200 nM) [63,64]. HOTAIR-PRC2 interactions might be very different from those of *Xist-*PRC1/2 **(**figure 1*b*). The interaction between HOTAIR and PRC2 is likely sustained by the repetitive Guanine stretches (G-tracts) found in the D1 helix [64]. This interpretation is in line with data from Somarowth *et al*. showing equal affinity of the PRC2 complex to natively purified or refolded HOTAIR 5′/3′ using *in vitro* assays [53]*.* Noticeably, the putative Xist-PRC2 interaction region (A-repeats) is missing the key RNA recognition sequences needed for specific interactions (discussed below) [65].

## 3. *Xist* and *HOTAIR* show different modes of interactions with polycomb-repressive complexes 1/2 components

**Figure 2.**
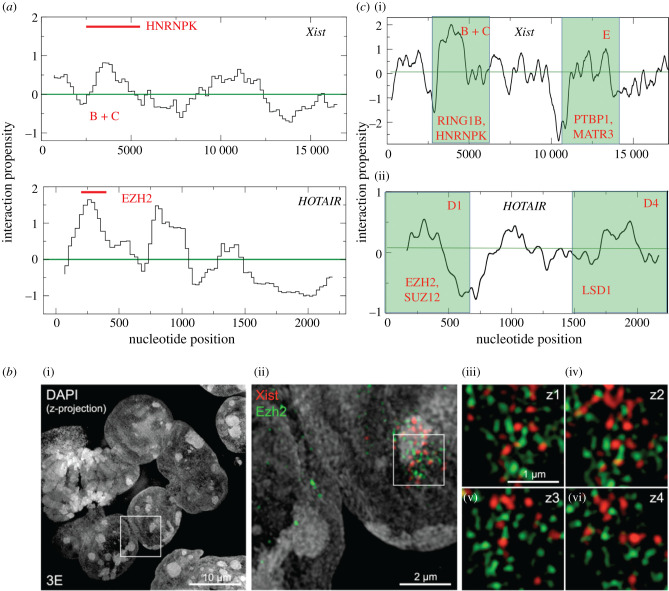
*Xist and HOTAIR RNA predicted structure and interaction propensity and super-resolution microscopy*. (*a*). *Xist* interaction propensity profile (Z-normalized binding propensities of RNA regions) calculated with *cat*RAPID indicates that the binding of HNRNPK is in the region comprising the Xist Rep B and the Xist Rep C [35], in agreement with experimental evidence [54]. (*b*) *Xist* and PRC2 do not directly interact. Representative image of *Xist* and PRC2 catalytic subunit Ezh2 from Cerase *et al.* [57]. Reproduced with the permission of the editor, PNAS February 11, 2014 111 (6) 2235–2240. (*c*) *HOTAIR* interaction propensity calculated with *cat*RAPID indicates the binding of EZH2 in the D1 domain [66], in agreement with experimental evidence [57,58]_._

We analysed our previously published data on *Xist* and *HOTAIR* [35,66,67] binding abilities to PRC1/2 components. In our studies, we employed the *cat*RAPID [35,68] method to estimates the binding potential of proteins to RNA molecules through van der Waals, hydrogen bonding and secondary structure propensities of both protein and RNA sequences. This allows the identification of binding partners with high confidence [69]. In agreement with experimental evidence [54], *cat*RAPID identified a direct interaction between *Xist* 5′-end and HNRNPK [35] (*Global Score* = 0.99 on a scale ranging from 0 to 1, where 0 indicates no RNA-binding ability and 1 strong affinity; figure 2*a*; by contrast, the negative control Dyskerin Pseudouridine Synthase 1 DKC1 has a score of 0.01). To identify interactions of long non-coding RNAs such as *Xist*, *cat*RAPID exploits a special pipeline that is based on the division of the transcript into fragments and calculation of their individual binding propensities (Z-normalized to 0 mean and standard deviation of 1), which is useful to spot the binding sites (figure 2*a*) [35]. PRC1 catalytic subunits Ring1A/B showed a high *cat*RAPID score (*Global Score* = 0.98) [35], in accordance with what reported by Chu *et al.* [39] using *Xist* complementary oligo probes in pull-down experiments. Yet, it should be mentioned that Chu *et al.* [39] used formaldehyde fixation conditions to identify *Xist* binders, which indicates that non-direct interactions can be detected in their experiments. Other PRC1 components and PRC2 subunits did not rank high in our *cat*RAPID analysis [35]. This is in agreement with the observation that PRC2 elements are under-represented in proteomic [36,37,39] and genetic screens [33,34] designed to reveal *Xist* interactomes. As for other PRC1 and PRC2 elements, we predicted low interaction propensities. For example, SUZ12, EZH1 and EZH2 have *Global Score* values of 0.01, 0.22 and 0.35, respectively. This is in line with the results of the previous analysis [70]. In brief, using randomized Xist A-repeats as a control, Ezh2 has been predicted to bind *Xist* with low affinity (EZH2-A-repeats interaction propensity is approximately 1, using a scale where positive interactions have scores greater than 10). These findings are in good agreement with three-dimensional-SIM data (figure 2*b*), showing the poor overlap between *Xist* and PRC2 [57], suggesting that this interaction might be sustained by intermediary proteins or via an indirect cascade (i.e. through PRC1-mediated H2A119 ubiquitination, see below).

On the other hand*, cat*RAPID predictions indicate that *HOTAIR* and EZH2 might directly interact (*Global Score* = 0.99; figure 2*a*; by contrast, the negative control, the keratin-associated protein KRTAP21 has a score of 0.01, which is in agreement with previous biochemical evidence [63,64]). In both *Xist* and *HOTAIR* analyses, protein interactions strictly occur in highly structured regions of the transcripts (figure 2*c*) that contain G-rich stretches. These findings are in line with recent studies revealing that double-stranded regions in RNA molecules provide the scaffold for protein complexes [71,72]. Indeed, since RNA transcripts are highly flexible, an increase in secondary structure makes the protein partners bind tightly [72], favouring their accumulation on the scaffold, which can induce the formation of phase-separated assemblies (discussed below) [71].

In regards to the RNA structure, the *CROSS* (Computational Recognition of Secondary Structure) algorithm predicts the propensity of a nucleotide to be double-stranded given the neighbour nucleotides and the crowded cellular environment [73]. *CROSS* has been previously employed to compute the structural properties of *Xist* and *HOTAIR* [73,74]. In accordance with dimethyl sulfate (DMS)-sensitivity experiments [75], CROSS [73] analysis predicts that, Xist B and C Repeats (nucleotides approximately 2000–5500) as well as Xist A repeats (nucleotides approximately 1–400) and E (nucleotides approximately 10 000–12 000) of *Xist* are highly structured. Among *Xist*-interacting proteins binding to RepE, there are the splicing regulators polypyrimidine Tract Binding Protein 1 (PTBP1), MATRIN-3 (MATR3), CUG-Binding Protein 1 (CELF1) and TAR-DNA Binding Protein (TDP-43) [35–37,39].

In the case of *HOTAIR,* CROSS [73] identifies specific regions in the D1 region (nucleotides 1–500) as the most structured, together with the adjacent D2 region (nucleotides 500–1000 and nucleotides 1500–2200), which agrees with DMS experiments [53] (figure 2*c*, bottom). In addition to EZH2 [76], HOTAIR was shown to interact with the histone demethylases LSD1 (lysine-specific demethylase 1A). LSD1 is a flavin-dependent monoamine oxidase that demethylates lysines, specifically lysine 4 on histone H3. LSD1 is known to form a multi-protein complex with REST (RE1-Silencing Transcription factor) and CoREST that are critical players in gene silencing [63,64].

## 4. Polycomb-repressive complexes 1/2–long non-coding RNA interactions and phase separation

Phase separation is defined as the process by which a homogeneous solution divides in two or more separated phases. Paraspeckles are a classic example of phase-separated cellular entities, *nucleoli* and stress granules [19,44–49], which are membrane-less assemblies composed of RNA and proteins. Formation of cytoplasmic stress granules is an evolutionary conserved mechanism. For example, stress granules are formed in response to environmental changes (i.e. heat shock) and favour the confinement of enzymes and nucleic acids in discrete regions of the nucleus or cytoplasm [77]. Structurally disordered and nucleic acid binding domains promote protein–protein and protein–RNA interactions in large ‘higher-order’ assemblies [78,79]. Intrinsically disordered proteins, which are enriched in polar and non-polar amino acids such as arginine and phenylalanine, have been shown to promote phase transitions in the cell [45].

**Figure 3.**
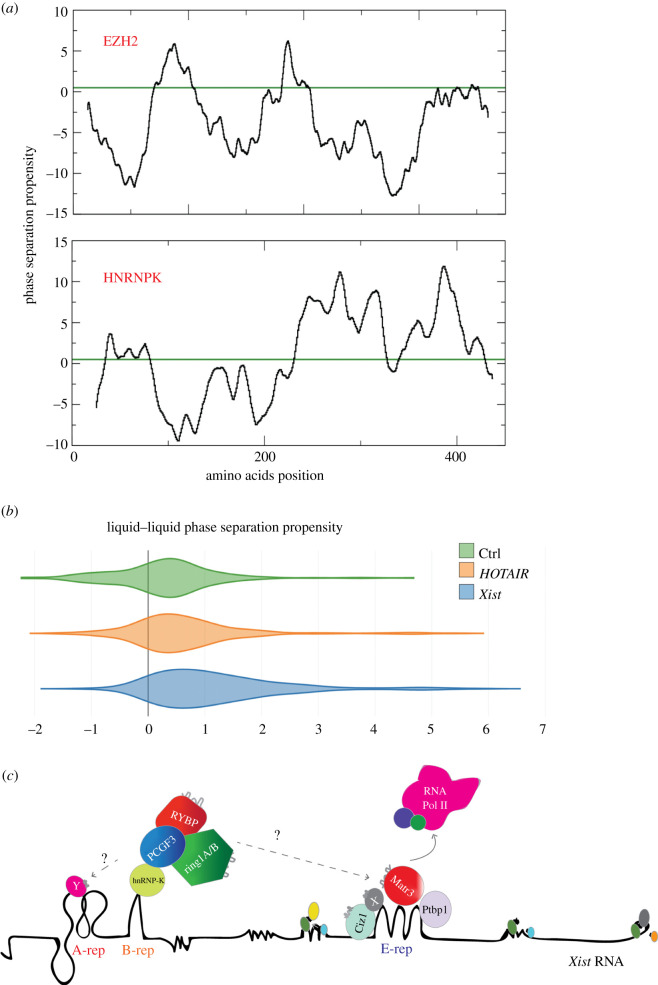
*Xist and HOTAIR phase separation propensity and phase separation by Xist RNA through recruitment of phase-separating proteins.* (*a*) Phase separation propensities profiles reveal that structurally disordered regions in EZH2 (https://pfam.xfam.org/protein/Q15910) and HNRNPK (https://pfam.xfam.org/protein/P61979) promote the formation of high-order assemblies. HNRNPK shows higher phase separation propensity than EZH2. (*b*) Comparison between *Xist* and *HOTAIR* interactomes indicates that *Xist* interactions are enriched in elements prone to phase separation (***p-value < 0.001; Kolmogorov–Smirnov test, table 1). Comparison with control RNA (antisense of the 3′ UTR of Alpha Synuclein) [80] indicates that *HOTAIR* has non-negligible propensity to associate with phase-separating proteins (***p-value < 0.001; Kolmogorov–Smirnov test). (*c*) The most-likely Xist-mediated PRC2 recruitment pathway involves PRC1 recruitment via repeat B interaction through HNRNPK direct interaction (light green). H2A ubiquitination by PRC1 may induce PRC2 recruitment on the Xi as previously shown (see main text). We suggest that *Xist* might also recruit PRC1/2 complexes by phase separation through mediation of structurally disordered proteins the Xist binding repeat E. Phase-separated PRC1/2 recruitment could occur through a direct interaction with repeat Xist E. We suggest that the PRC1/2 oligomerization can further recruit repressive proteins and/or disordered proteins, contributing to the eviction of Pol II and basic transcription factors, recruiting more structurally disordered proteins and in turn, inducing further granule formation, heterochromatinization and gene repression. Xist repeats are shown; A repeat (pink), B repeat (orange); E repeat (blue). Proteins are shown by name. Waved grey profiles on proteins, indicate intrinsically disordered regions; Xist RNA (black line).

In a recent publication [67], we reasoned that *Xist* exerts its functions—at least in part—through the formation of silencing granules by phase separation, in which PRC1 and PRC2 are also recruited. More precisely, we suggested that non-canonical recruitment of repressive PRC1 complexes is promoted or reinforced by the formation of higher order assemblies. In this scenario, the primary *de novo* recruitment of PRC1/2 would happen through the *Xist* B repeats [54] direct interaction and involve proteins with a strong propensity to phase separate. As predicted by the *cat*GRANULE algorithm [45] that estimates the ability of proteins to form liquid-like assemblies containing protein and RNA molecules [67], both EZH2 and HNRNPK are prone to phase-separate (figure 3*a* and table 1). Yet, HNRNPK shows a much higher granulation score than EZH2 (1.60 versus 0.71; note that the score is z-normalized and 0 correspond to the average protein propensity), which suggests enhanced ability to form large ribonucleoprotein complexes. In agreement with this observation, experimental [57,81] and computational studies [67] have indicated that *Xist* could phase separate with its associated proteins, but no evidence has been proposed so far on *HOTAIR* ability to form such assemblies. This finding is in line with the fact that PRC2 components might directly binding to *HOTAIR*, while most of *Xist-*Polycomb associations [51,82] are largely indirect [54] (figures 1 and 2). Indeed, analysing the whole protein interactomes of both *Xist* [36,39] and *HOTAIR* [83], we found that *Xist* binding partners are highly prone to phase separation, while *HOTAIR* interactions show lower propensity to phase separate, which is in accordance with the observation that indirect protein–protein interactions may mediate associations through structurally disordered domains (figure 3*b***)** [67]. We note that *HOTAIR* binding partners have a non-negligible propensity to phase separate with respect to a similar length negative control (antisense of 3′ UTR of Alpha Synuclein; around 2500 nucleotides; figure 3*b*) [80], which suggests that *HOTAIR* might form medium-size assemblies [84].

**Table 1.** Liquid–liquid Phase Separation (LLPS) propensity of PRC1 and PRC2 components. The score is Z-normalized and values >0 indicate that the protein is prone to phase-separate.

gene	LLPS
HNRNPK	1.601
RING1	1.499
JARD2	1.339
SUZ12	1.226
CBX2	1.175
CBX4	1.169
CBX8	1.058
EZH2	0.711
CBX6	0.592
PHC1	0.556
EED	0.509
RBBP4	0.466
PHC2	0.401
RING2	0.107
BMI1	−0.059
PCGF2	−0.096
PHC3	−0.438
CBX7	−0.439

In the *Xist* case, PRC1 positive feedback recruitment may be reinforced by liquid-like interactions in which specific elements such as CBX2 [85] (liquid–liquid phase separation propensities of 1.17 [45]) as well as SAM-domain multimerization [86] or intrinsically disordered domains could be involved. Based on their phase separation scores, we speculate that other proteins such as HNRNPU (phase separation propensity of 2.5) and MATR3 (liquid–liquid phase separation propensity of 1.5) might contribute towards the recruitment of polycomb proteins to the *Xist* body (table 1). These interactions might also be mediated by intrinsically disordered proteins yet to be discovered binding the Xist A-, D-3′end repeats. This protein multimerization driven by phase separation and the RNA–protein interactions might be playing a critical role in this process [67] and, in turn, trigger RNA Polymerase II (Pol-II) and basic transcription factors eviction, inducing gene silencing and heterochromatinization (figure 3*c*).

## 5. Non-catalytic functions of polycomb-repressive complex in shaping the three-dimensional genome might be mediated by RNA interactions

**Figure 4.**
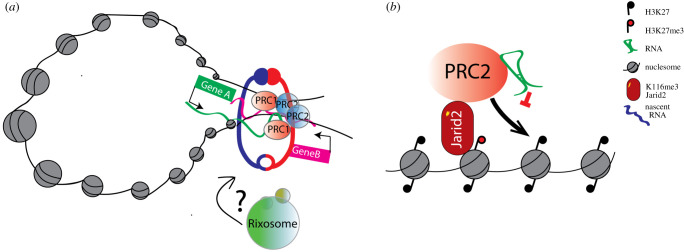
*RNA sustains Polycomb complexes functions*. RNA can facilitate PRC1/2 complex and sustain three-dimensional contacts and loops (also mediated by the cohesin complex; red/blue ring) to coordinate gene expression by brining co-regulated genes together (gene A, green; Gene B, purple; green/blue ribbons represent nascent RNA from gene A/B). Rixosome could also be participating to these interactions. (B) *RNA inhibits PRC2 catalytic activity.* RNA (green) can inhibit PRC2 catalytic activity. Its activity can be relieved by H3K27me3 tails (red lollipop) or methylated Jarid2 proteins.

Work from different laboratories has shown that PRC1/2 complexes are essential regulators of cellular three-dimensional structure (recently reviewed by Illingworth RS [85] and Cheutin and Cavalli [87]). Very recent work from the Cavalli lab has elegantly shown how PRC1 can exert different and apparently opposing functions such as gene repression, three-dimensional organization of the genome and gene activation [88]. In brief, Loubiere and colleagues showed, using PRC1 mutants at the *duchsund* locus in Drosophila, that genes are positively and negatively regulated by PRC1. In particular, they suggest that while in the absence of activating transcription factors (TFs), PRC1 is mostly involved in gene silencing, in the presence of TF, PRC1 might be able to regulate gene expression by making PRC1-dependent promoter enhancer contacts [88]. As PRC1 has also been shown to have a role in regulating occupancy, elongation and phosphorylation of RNA polymerase II (Pol-II) [89,90], it is tempting to speculate that these functions of PRC1 might be, in part, mediated by its ability to bind to RNA via RING1A/B or CBX7 [91] proteins (figure 4*a*). In support of this idea/interpretation, a paper from the Moazed laboratory [92] has shown that the Rixosome, a conserved RNA degradation machinery, interacts with PRC1/2, and it is recruited at Polycomb sites for efficient gene silencing. Similarly, Garland et al. [93] showed a link between the RNA degradation pathways and Polycomb silencing. In particular, they showed that KO of Zcfh31, a component of the poly(A) RNA exosome targeting (PAXT) complex, increases the cellular level of poly-adenylated RNA, triggering the destabilization of the PRC2 complex, impaired chromatin binding and reduction of gene silencing [93]. Furthermore, work from several laboratories has shown that Polycomb can interact with RNAs [94,95], nascent transcripts [96] or with R-loops at Polycomb-repressed targets [94,97]. These lines of evidence support the idea that the interaction of Polycomb proteins with RNA might be spurious, yet it is critical for numerous cellular functions, from nuclear three-dimensional organization [85,87,98–100], repression of target genes [94,101,102], spreading on PRC1/2 [103], cellular differentiation and lineage commitment.

## 6. Conclusion

Elegant biochemistry work from several laboratories showed that PRC1 [19] and specific PRC2 subcomplexes [20,104] (i.e. PRC2.1, PRC2.2 depending on the accessory subunits present in the complex, reviewed in Van Mierlo and colleagues [9]) bind to RNA with different affinities and specificities. Recent work suggests that the interaction of PRC1/2 components to RNA is promiscuous [18,105], and in part mediated by protein–protein interactions [65]. It has also been shown that EZH2–RNA interactions can catalytically inactivate or expel EZH2 [101,104,106–108], suggesting that RNA binding is essential for the modulation of polycomb catalytic activities [50] (figure 4*b*). However, allosteric RNA inhibition can be relieved both by H3K27me3 and methylated JARID protein interactions (the latter also in agreement with Cifuentes-Rojas and colleagues [106,109]). These lines of evidence suggest a new model of PRC2 recruitment that can explain both *de novo* polycomb recruitment (RNA binding) and spreading (using established polycomb domains) [20].

Taking into account previous experimental and computational work, we suggest that the ‘canonical’, direct lncRNA-mediated PRC2 recruitment has to be revisited [105]. As for *Xist*, the *de novo* recruitment of PRC1 and PRC2 is highly unlikely to occur through a mechanism of recruitment to the chromatin associated with catalytically inactivated complexes (i.e. allosteric inhibition). Although the recruitment of *Xist* to pre-existing CpG islands might partially alleviate its catalytic inhibition (104). Alternatively, these interactions occur indirectly (no complex inhibition), through intermediate proteins or by means of liquid–liquid phase separation (figure 3*a–c*). For example, *Xist* A-repeats, the putative Xist-PRC2 interaction region, are missing the key RNA recognition sequences needed for specific interactions [65], which suggests that these interactions, although critical, might also be spurious [18,65,105] (binding many RNAs with low affinity) or indirect. As for the HOTAIR-mediated *de novo* Polycomb recruitment (possibly mediated by direct interactions), it is possible that residual H3K27me3 at the HOX locus might alleviate allosteric inhibition [110]. For PRC1/2 recruitment on the inactive X chromosome (Xi) at the onset of XCI, it is likely that *de novo* accumulation largely depends on PRC1-mediated mark on the chromatin, such as H2A-119ub (figure 1*a*) [23,54,58,104]. In this regard, work from the Pasini and Klose laboratory elegantly proved that H2A119 ubiquitination is essential for PRC1/2 silencing and PRC2 *de novo* recruitment [22,23,59]. We believe that more work has to be done in order to have a final model of lncRNA and Polycomb recruitment, capable of reconciling all this evidence.

## 7. Material and methods

### 7.1. RNA–protein interaction predictions and granule propensity

To compute protein-RNA interactions, we used the catRAPID approach that evaluates the interaction propensities of polypeptide and nucleotide chains based on their physico-chemical properties predicted from primary structure [35,66]. Structural disorder, nucleic acid-binding propensity and amino acid patterns such as arginine-glycine and phenylalanine-glycine are key features of proteins coalescing in granules [45]. These features were combined in a computational approach, *cat*GRANULE, that we employed to identify RBPs assembling into granules (scores >0 indicate granule propensity). We predicted the secondary structure of transcripts using CROSS [73,74]. The algorithm predicts the structural profile (single- and double-stranded state) at single-nucleotide resolution using sequence information only and without sequence length restrictions (scores > 0 indicate double stranded regions).

HOTAIR repeats annotation: D1 (nucleotides 1–530) consists of 12 helices, 8 terminal loops and 4 junctions (three 3-way junctions and one 4-way junction). D2 (nucleotides 531–1040) consists of 15 helices, 11 terminal loops and 4 junctions (three 5-way junctions and one 3-way junction). D3 (nucleotides 1041–1513) is the smallest of all the four domains and consists of 9 helices, 6 terminal loops and 3 junctions (two 4-way junctions and one 3-way junction). Finally, D4 (nucleotides 1514–2148) is the largest among the four domains and consists of 20 helices, 13 terminal loops and 7 junctions (one 6-way, two 4-way and four 3-way junctions).

## Supplementary Material

short summary

## Data accessibility

Relevant data are available at 1) http://crg-webservice.s3.amazonaws.com/submissions/2020-03/251545/output/index.html?unlock=073199e0ac (Ezh2-Hotair); http://crg-webservice.s3.amazonaws.com/submissions/2020-03/251800/output/index.html?unlock=6cbd243faa (HnrnpK-Xist); http://crg-webservice.s3.amazonaws.com/submissions/2020-03/251574/output/index.html?unlock=c891ca7c43 (Xist); http://crg-webservice.s3.amazonaws.com/submissions/2020-03/251583/output/index.html?unlock=0c097f6326 (Hotair).
