# Supplementary material for: Long non-coding RNA-polycomb intimate rendezvous
Source: Open Biol. 2020 Sep 9;10(9):200126. doi: 10.1098/rsob.200126 (PMC7536065; doi:10.1098/rsob.200126)
Supplement: short summary [file rsob200126supp1.docx]

4) A media summary: a short non-technical summary (up to 100 words) of the key findings/importance of your manuscript. Please try to write in simple English, avoid jargon, explain the importance of the topic, outline the main implications and describe why this topic is newsworthy.

In this review, we critically analyze the interaction of Polycomb complexes with two long non-coding RNA, Xist and HOTAIR, suggesting similarities and differences in their way of action. In particular, we summarize the current literature on Polycomb repressive complexes regarding their ability to bind RNA. While these interactions have been previously regarded as spurious and mostly regulating the catalytic activity of these complexes, we here suggest that these interactions are critical for the biology of the cell. We suggest that these interactions are crucial for 3D-genome organization, RNA-mediated gene regulation and sustain phase separation.
